# A Lateral Flow Immunoassay for the Rapid Identification of CTX-M-Producing Enterobacterales from Culture Plates and Positive Blood Cultures

**DOI:** 10.3390/diagnostics10100764

**Published:** 2020-09-28

**Authors:** Sandrine Bernabeu, Kayaththiry Caroline Ratnam, Hervé Boutal, Camille Gonzalez, Anaïs Vogel, Karine Devilliers, Marc Plaisance, Saoussen Oueslati, Surbhi Malhotra-Kumar, Laurent Dortet, Nicolas Fortineau, Stéphanie Simon, Hervé Volland, Thierry Naas

**Affiliations:** 1 Team Resist, UMR1184, School of Medicine of Université Paris-Saclay—INSERM—CEA, LabEx Lermit, 94276 Le Kremlin-Bicêtre, France; sandrine.bernabeu@aphp.fr (S.B.); oueslati.saoussen@gmail.com (S.O.); laurent.dortet@aphp.fr (L.D.); nicolas.fortineau@aphp.fr (N.F.); 2 Bacteriology-Hygiene Unit, APHP, Hôpital Bicêtre, 94270 Le Kremlin-Bicêtre, France; ratnam.caroline@gmail.com (K.C.R.); gonzalezcamille0405@gmail.com (C.G.); 3 Service de Pharmacologie et Immunoanalyse (SPI), CEA, INRA, Laboratoire d’Etudes et de Recherches en Immunonalyse, Université Paris-Saclay, 91191 Gif-sur-Yvette, France; Herve.BOUTAL@cea.fr (H.B.); anais.vogel@cea.fr (A.V.); karine.devilliers@cea.fr (K.D.); marc.plaisance@cea.fr (M.P.); stephanie.simon@cea.fr (S.S.); herve.volland@cea.fr (H.V.); 4 Laboratory of Medical Microbiology, Vaccine & Infectious Disease Institute, University of Antwerp, 2610 Antwerp, Belgium; surbhi.malhotra@uantwerpen.be; 5 Members of ESCMID Study Group for Antimicrobial Resistance Surveillance—ESGARS, Headquarter, 4010 Basel, Switzerland; 6 Associated French National Reference Center for Antibiotic Resistance: Carbapenemase-Producing Enterobacteriaceae, 94270 Le Kremlin-Bicêtre, France; 7 Service de Bactériologie, AP-HP, CHU de Bicêtre, 78 Rue du Général Leclerc, 94275 Le Kremlin-Bicêtre, France

**Keywords:** CTX-M, ESBL, LFIA, rapid detection, blood culture

## Abstract

We have developed a lateral flow immunoassay (LFIA), named NG-Test CTX-M MULTI (NG-Test), to detect group 1, 2, 8, 9, 25 CTX-M producers from agar plates and from positive blood cultures in less than 15 min. The NG-Test was validated retrospectively on 113 well-characterized enterobacterial isolates, prospectively on 102 consecutively isolated ESBL-producers from the Bicêtre hospital and on 100 consecutive blood cultures positive with a gram-negative bacilli (GNB). The NG-Test was able to detect all CTX-M producers grown on the different agar plates used in clinical microbiology laboratories. No false positive nor negative results were observed. Among the 102 consecutive ESBL isolates, three hyper mucous isolates showed an incorrect migration leading to invalid results (no control band). Using an adapted protocol, the results could be validated. The NG-Test detected 99/102 ESBLs as being CTX-Ms. Three SHV producers were not detected. Among the 100 positive blood cultures with GNB tested 10/11 ESBL-producers were detected (8 CTX-M-15, 2 CTX-M-27). One SHV-2-producing-*E. cloacae* was missed. The NG-Test CTX-M MULTI showed 100% sensitivity and specificity with isolates cultured on agar plates and was able to detect 98% of the ESBL-producers identified in our clinical setting either from colonies or from positive blood cultures.

## 1. Introduction

The production of β-lactamases is a commonly encountered mechanism of resistance in Gram-negative bacteria, especially Enterobacterales [1]. In healthcare settings, extended-spectrum-β-lactamase (ESBL)-producing Enterobacterales (ESBL-E) are of particular concern. The CDC has estimated that ESBL-Es account for 19% of healthcare-related infections annually and that infections involving by these bacteria are also associated with increased mortality and cost of care [2]. ESBLs limit treatment options because they hydrolyze most β-lactams, including penicillins and third-generation cephalosporins (e.g., ceftazidime), and carry many other resistance determinants leading to a multidrug-resistant (MDR) phenotype [1].

Within the ESBLs, the CTX-M family appeared in the early 80 s and represent now the most prevalent ESBLs worldwide. They are divided into five groups based on amino acid sequence identity: the CTX-M-1, CTX-M-2, CTX-M-8, CTX-M-9, and CTX-M-25 groups. In each group are included minor allelic variants differing one from the other by one or few amino-acid substitutions [3,4,5]. Since the first description, they disseminated worldwide, becoming the most prevalent ESBLs [6,7]. The CTX-M-15 variant from the CTX-M-1 group is considered as the most prevalent one in many parts of the world. It is supplanted by CTX-M-9-group variants in China, Japan, South-East Asia and Spain for example, where the variant CTX-M-14 is dominant [7]. They have disseminated among a wide range of clinical bacteria species. Although they are susceptible to inhibitors, infections caused by CTX-M-producing bacteria present limited options of treatments. This situation has led to an increasing use of carbapenems, followed by the emergence and spread of carbapenemase-producing strains [7,8].

Enterobacterales expressing CTX-Ms are associated with higher rates of healthcare costs, morbidity and mortality [7,8,9,10,11,12]. Infection control strategies for ESBL-Es are based on rapid identification of carriers. In addition, infections caused by ESBL-Es present limited treatment options, thus leading to an increasing use of carbapenems as observed in many countries [13]. Several diagnostic tests already exist for the identification of CTX-M carriers in clinical setups. They are mainly based on phenotypic approaches with antimicrobial susceptibility using disc diffusion methods in presence and absence of β-lactamase inhibitors [14,15]. These conventional screenings are usually slow, requiring an over-night culture (o/n), are labor-intense and require expertise for interpretation. The molecular methods, either DNA microarray-based and/or nucleic acid amplification-based remain the gold standard for the detection of CTX-Ms and other ESBLs [14,15,16,17,18,19,20,21,22]. Many of these assays correspond to home-made PCR assays [19,20], but several assays are commercially available for CTX-M detection alone [18], and others for detecting also the 5 main carbapenemases such as the Verigene^®^ system (Luminex Corporation, Austin, TX, USA) and the FilmArray^®^ system (bioMérieux, Marcy l’Etoile, France). The two latter may be used directly from patients’ positive blood cultures [21,22]. However, these tests are usually slow (1–4 h), labor-intense, expensive, require specific equipment and trained staff in order to be implemented in any clinical laboratory. To meet current needs, antimicrobial drug resistance detection methods must be cheap (reduced cost of consumables and equipment) and easy to use (reduce technical complexity) for the end user. Lateral flow immunoassays (LFIAs) have shown their usefulness as confirmatory tests for antibiotic resistance mechanisms detection, especially for β-lactamases in gram-negatives [23,24,25]. They provide easy, rapid, and reliable results in a few minutes directly on colonies isolated on selective media or disk diffusion antibiogram [23,24,25]. The aim of the study was to develop a rapid LFIA, named NG-Test CTX-M MULTI, for the detection of the CTX-Ms belonging to the five groups directly from colonies isolated on different culture media widely used in routine laboratory and from positive blood cultures.

Here, we validated the NG-Test CTX-M MULTI LFIA retrospectively on 113 well-characterized enterobacterial isolates, and prospectively on 102 consecutively isolated ESBL-producers and on 100 consecutive blood cultures positive with a gram-negative bacilli (GNB). All the isolates expressing a CTX-M where perfectly detected within 15 min, showing 100% sensitivity and specificity for the 5-groups of CTX-M β-lactamases.

## 2. Materials and Methods

### 2.1. Ethics Statement

All experiments were performed in compliance with French and European regulations on the care of laboratory animals (European Community [EC] Directive 86/609, French Law 2001-486, 6 June 2001) and with the agreements (approved 6 January 2016) of the ethics committee of the Commissariat à l’Energie Atomique (CEtEA “Comité d’Ethique en Expérimentation Animale” n° 44) no. 12-026 and 15-055 delivered to S. Simon by the French Veterinary Services and CEA agreement D-91-272-106 from the Veterinary Inspection Department of Essonne (France).

### 2.2. Monoclonal Antibodies

Biozzi mice (10 weeks old) were immunized with the recombinant CTX-M-15 (M1-L291), CTX-M-2 (Q29-F291), and CTX-M-14 (Q29-L291) β-lactamases, as they are the most prevalent variants belonging to the three main groups of CTX-Ms (group 2, 9 and 1, respectively). These enzymes were expressed with the hexa-histidine tag on their C terminus as previously described [24,25]. For each enzyme, 20 monoclonal antibodies (mAbs) were tested, and the pairs of antibodies showing the best limit of detection (LOD) for CTX-M-expressing bacteria were selected for the NG-Test CTX-M MULTI assay as previously described [24,25]. The 3 best pairs of monoclonal antibodies selected (one against each targeted group), when combined in a single test line, were able to detect all 5 groups of CTXMs due to cross-reactivity of some antibodies with the two other groups (group 8 and 25).

### 2.3. NG-Test CTX-M MULTI Assay and Protocol

**Figure 1:**
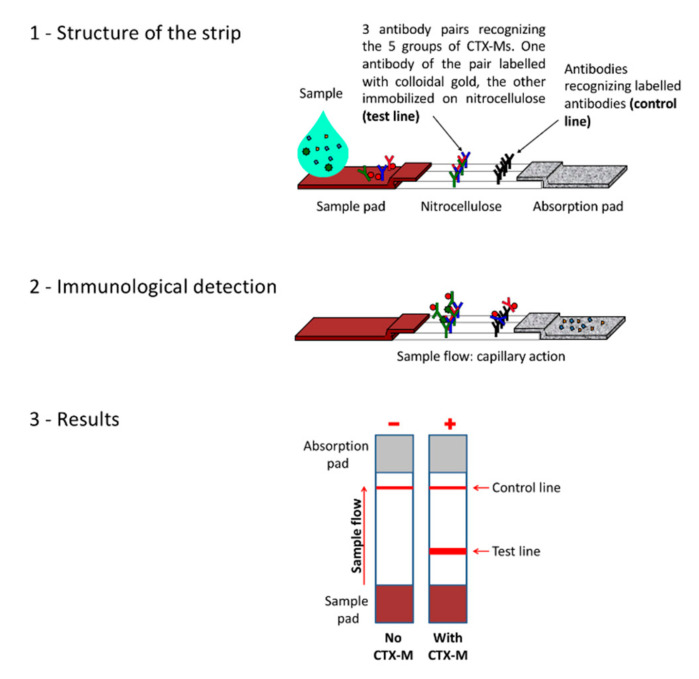
Principle of the assay.

The selected antibodies were produced on a large scale and provided to NG biotech (Guipry, France) for the development of the NG-Test CTX-M MULTI assay (Figure 1). Capture antibodies were immobilized on a unique test line. Isolates to be tested were grown overnight at 37 °C on MH agar plates. Using a 1 µL inoculation loop, a single colony was resuspended in 5 drops (c.a. 100 µL) of the extraction buffer (lysis step) and vortexed a few seconds. Subsequently, 100 µL of this extract were dispensed on the cassette, and the migration was allowed for 15 min. The results were eye read by monitoring the appearance of a red band on the test line, along with a band corresponding to the internal control.

### 2.4. Bacterial Strains

For the retrospective validation, 113 enterobacterial isolates with PCR-characterized β-lactamase content were used to evaluate NG-Test CTX-M MULTI. This collection represented 13 non-CTX-M ESBL-producers, 51 CTX-M ESBL-producers, 28 *Kluyvera* spp and 21 *Klebsiella oxytoca* isolates. Twenty-seven different CTX-M variants were tested (CTX-M-1, -2, -3, -8, -9, -10, -13, -14, -15, -17 -18, -19, -24, -27, -32, -37, -55, -57, -65, -71, -82, -93, -94, -100, -101, -127, and 182) belonging to the CTX-M-1 group (*n* = 32), CTX-M-2 group (*n* = 2), CTX-M-8 group (*n* = 2), CTX-M-9 group (*n* = 13) and CTX-M-25 group (*n* = 2).

For the prospective evaluation, 102 enterobacterial clinical isolates showing an antibiotic susceptibility profile compatible with the expression of an ESBL (Resistance to expanded-cephalosporins, recovery with class A inhibitors, with or without a synergy image), were tested from MH-agar plates.

### 2.5. Positive Blood Cultures, Isolate Identification and Susceptibility Testing

100 consecutive blood cultures positive with Gram-negative bacteria as revealed by Gram staining collected between November 2018 and April 2019 were directly tested according to the protocol described by Takissian et al. [23]. Bacteria recovered from these blood cultures were identified using matrix-assisted laser desorption ionization–time of flight mass spectrometry (MALDI-TOF MS) with the Bruker MS system (Bruker Daltonics, Bremen, Germany), according to the manufacturer’s instructions. Susceptibility testing was performed by the disk diffusion method on Mueller-Hinton agar plates (Bio-Rad, Marnes la Coquette, France) incubated for 18 h at 37 °C and interpreted according to EUCAST guidelines [26].

### 2.6. Resistance Gene Detection

Genes coding for Ambler class A β-lactamases were sought by PCR using primers specific for the *bla*_TEM_, *bla*_SHV_, and *bla*_CTX-M_ genes, as previously described [19,20]. PCR products were purified using the QIAquick PCR purification kit (Qiagen, Courtabœuf, France) and sequenced on both strands with an automated sequencer (ABI 3100; Applied Biosystems, Foster City, CA, USA).

### 2.7. Culture Media Tested

Eleven representative isolates were grown on 5 commonly used culture media: Mueller-Hinton (MH) agar (Biorad, Marnes la Coquette, France), URISelect 4 (Uri-4; Biorad, Marnes la Coquette, France), Columbia agar plus 5% horse blood (COH; bioMérieux, Marcy l’Etoile, France), ChromID ESBL agar (bioMérieux, Marcy l’Etoile, France) and Drigalski agar (bioMérieux, Marcy l’Etoile, France).

## 3. Results

### 3.1. Retrospective Evaluation on Well-Characterized Isolates

**Figure 2:**
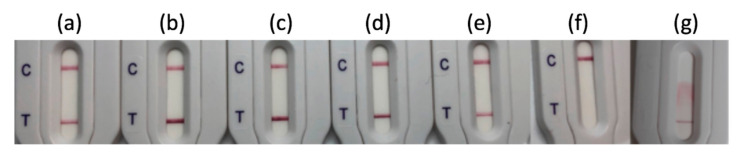
NG-Test CTX-M-MULTI results obtained with (**a**) E. coli expressing CTX-M-15; (**b**) E. coli expressing CTX-M-2; (**c**) E. coli expressing CTX-M-8; (**d**) E. coli expressing CTX-M-14; (**e**) E. coli expressing CTX-M-100; (**f**) E. cloacae expressing SHV-12 and (**g**) K. pneumoniae expressing CTX-M-18 that yeilded an invalid test result. One colony resuspended in extraction buffer and loaded on the cassette as recommended by the manufacturer. C stands for control line and T for test line.

NG-Test CTX-M-MULTI was able to detect unambiguously all the 51 CTX-M-producing isolates including 32 CTX-M-1-group (i.e., CTX-M-1, -3, -10, -15, -32, -37, -55, -57, -71, -82, -101, -127 and -182 enzymes), 2 CTX-M-2, 2 CTX-M-8-group, 13 CTX-M-9-group (i.e., CTX-M-9, -13, -14, -17, -18, -19, -24, -27, -65 and -93 enzymes) and 2 CTX-M-25-group (e.g., CTX-M-94 and -100 enzymes) producers (Figure 2 and Table S1). The detection occurred equally well with the different Enterobacterales species tested (30/30 *E. coli*, 5/7 *K. pneumoniae*, 4/4 *E. cloacae*, 2/3 *C. freundii*, 2/2 *K. oxytoca*, 2/2 *P. mirabilis*, 1/1 *C. koseri*). For three mucoid bacterial isolates (2 *K. pneumoniae* and 1 *C. freundii*) an invalid test result was obtained, as revealed on the strip by the absence of a materialized control line (Figure 2g). For these isolates, one colony was resuspended in extraction buffer, vortexed vigorously for 3 min, and incubated for 10 min at room temperature prior to loading on the LFIA. Using this adapted protocol, the three isolates were efficiently detected as CTX-M positive. The 13 isolates expressing non-CTX-M ESBL including 3 SHV-like, 6 TEM-like, 1 VEB-like and 3 SHV-like and TEM- like co-producers gave negative results (Table S1).

**Figure 3:**
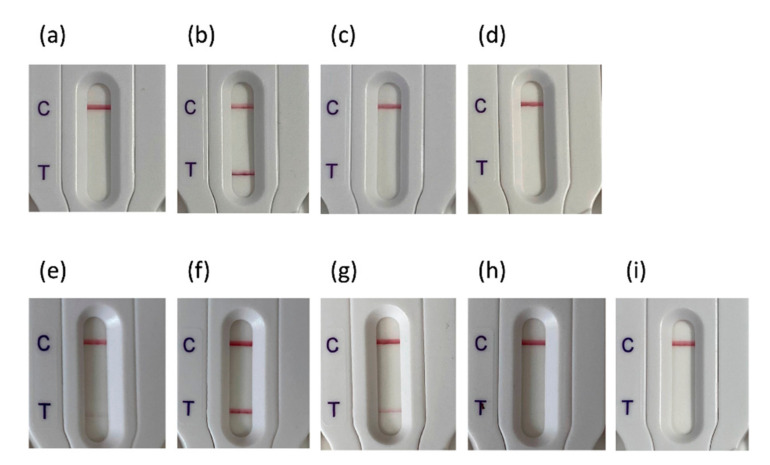
NG-Test CTX-M-MULTI results obtained with (**a**) K. oxytoca expressing NDM-1; (**b**) K. oxytoca expressing OXA-48, VIM-1 and CTX-M-15; (**c**) K. oxytoca WT; (**d**) K. oxytoca expressing TEM-1; (**e**) K. ascorbata CIP8295; (**f**) K. ascorbata (O23C8; patient); (**g**) K. ascorbata (O38I1, patient); (**h**) K. cryocrescens (CIP7952); (**i**) K. ascorbata (20-141, patient). One colony resuspended in extraction buffer and loaded on the cassette as recommended by the manufacturer. C stands for control line and T for test line.

As *K. oxytoca* produces a chromosomally encoded ß-lactamase that is closely related to CTX-Ms in terms of AA sequence identity (88% AA identity), we tested 21 clinical *K. oxytoca* isolates expressing various acquired ß-lactamases in addition to the endogenous OXY variant (Figure 3; Table S2). None of the *K. oxytoca* isolates tested gave a positive test result, except four isolates that produced an acquired plasmid-encoded CTX-M-15. Finally, as CTX-M enzymes derive from chromosomally borne β-lactamases from different *Kluyvera species*, 28 *Kluyvera* spp. isolates were tested, being either reference isolates of the Pasteur Institute strain depository (CIP) or clinical isolates from our strain collections. Thus 3 *K. cryocescens*, 1 *Kluyvera* sp., 3 *K. cochlae*, 1 *K. georginia*, and 20 *K. ascorbata* were tested (Figure 3; Table S3). One *K. ascorbata* gave a strong result, which is correlated with the high level of resistance to β-lactams of this isolate (Data not shown). Interestingly some isolates (8) had very faint bands, suggesting low level expression, while 19 gave a clear negative result, compatible with the fact that in *Kluyvera* spp., the chromosomally encoded CTX-M variants are weekly, if at all, expressed.

Taken together the NG-Test CTX-M MULTI is a highly sensitive and specific assay (as no false positive nor false negative results were observed) for CTX-M detection.

### 3.2. Evaluation of NG-Test CTX-M MULTI Results on Different Culture Media

Eleven isolates (2 CTX-M-1 group, 2 CTX-M-2-group, 2 CTX-M-8 and 2 CTX-M-9-group producers and 3 non-CTX-M producers) from the retrospective validation assay were grown on 5 of the most commonly used media for bacterial growth (Table 1). Some media currently used for the identification and/or selection of ESBL-expressing strains generate colonies with genus-specific colors (blue, green, pink, or dark purple on Uri-4 plates for example). These colored colonies, once suspended in the extraction buffer, stained the latter in a similar manner. This staining didn’t interfere with the results. The 8 CTX-M-expressing strains gave positive results and the 3 non-CTX-M-producers gave negative results. Colony staining didn’t change the appearance of the nitrocellulose membrane and still yielded easily interpretable results (data not shown).

### 3.3. Prospective Evaluation on Routine Antibiograms

For this study, 102 consecutive expanded-spectrum-cephalosporin-resistant Enterobacterales collected at the Bicêtre hospital from March to July 2018 were included in this study. These bacteria were recovered from urine (43%), from blood cultures (55%) and from a bile (2%) (Table 2). The most prevalent bacteria found in urine and in blood cultures were *E. coli* (71%), and *K. pneumoniae* (73%), respectively. Bacteria were directly tested from a disk diffusion antibiogram on MH agar. ESBL production was monitored by synergy testing, LFIA using the NG-Test CTX-M MULTI and by homemade-PCR sequencing [19,20]. Out of the 102 isolates compatible with ESBL production according to routine antibiogram, 99 were positive using the NG-Test CTX-M MULTI assay (Table 2). Three gave a negative test result, one SHV-12 producing *E. cloacae* and two hyper producing SHV-28 *K. pneumoniae* isolates. No invalid test result was observed, no false negative and no false positive results were observed. The results matched perfectly with the PCR-sequencing results. Eighty-one percentage of the ESBLs detected belonged to CTX-M-1 group with CTX-M-15 representing 72% of the total ESBLs. 16% of the ESBLs detected belonged to CTX-M-9 group and 3% were SHV-producers. No plasmid-encoded AmpC (pAmpC) enzymes were detected during this study.

### 3.4. Prospective Evaluation on Blood Culture

Among the 100 consecutive positive blood cultures with Gram negative Bacilli (GNB), as revealed by gram staining, the NG-Test CTX-M MULTI tested directly without prior sub-culturing using the published protocol by Takissian et al. [23] gave 10 positive results. Routine disk diffusion antibiograms revealed the presence of a phenotype compatible with ESBL production (resistance to expanded-spectrum cephalosporins and recovery by clavulanate) with 11 bacteria grown from these blood cultures. MALDI-TOF MS identification of the bacteria and PCR-sequencing revealed the presence of 5 CTX-M-15-producing *E. cloacae*, 3 CTX-M-15-producing *E. coli*, 2 CTX-M-27-producing *E. coli* and 1 SHV-12-producing *E. cloacae*). The 10 CTX-M-producing bacteria were from the 10 blood cultures identified as CTX-M-positive by the NG-Test CTX-M MULTI.

## 4. Discussion

CTX-M enzymes are today the most prevalent group of ESBLs among Gram-negative pathogens around the world, representing a global pandemic [7]. There are around 229 CTX-Ms variants described that belong to either CTX-M-1, -2, -8, -9 and -25 clusters differing in less than 5% amino acid sequence within each cluster [27,28], including all plasmid-encoded and the chromosomic variants in *Kluyvera* species [29]. The global epidemiology of CTX-M variants is complex with some regional specificities [7], but CTX-M-15 is now the most prevalent CTX-M determinant worldwide [7]. Other variants, such as group 9 variants (especially CTX-M-14) are prevalent in China, South-East Asia, South Korea, Japan and Spain, and group 2 and group 8 (CTX-M-2-like and CTX-M-8-like) variants are prevalent in South America [7]. In many European countries, such as in France CTX-M-15 has become the major ESBL encountered in clinical isolates [7]. In our study, 97% of the ESBLs were CTX-M-like enzymes and 3% SHVs (one SHV-12 producing *E. cloacae* and two hyper producing SHV-28 *K. pneumoniae* isolates). Eighty-one % of the ESBLs detected belonged to CTX-M-1 group with CTX-M-15 representing 72% of the total ESBLs. 16% of the ESBLs detected belonged to CTX-M-9 group and 3% were SHV-producers. No pAmpC enzymes were detected during this study, revealing the low prevalence of theses enzymes in our hospital.

As *bla*_CTX-M_ genes are encoded on multidrug-resistant plasmids, treatments of severe infections with CTX-M-producing bacteria rely on carbapenems and are associated with higher rates of healthcare costs, and mortality [1,12]. Rapid identification of CTX-M producers is mandatory for implementation of infection control measures and for initiating rapidly proper treatment [1,12]. Easy-to-carry-out tests, mostly based on inhibition of ESBLs by clavulanic acid or tazobactam, are recommended [30]. A new phenotypic assay that originates from the carbapenem inactivation method (CIM) [31], named the direct β-lactam inactivation method (dBLIM), that allows detection of extended-spectrum-cephalosporinase activity directly from Enterobacterales in culture or from positive blood cultures, using a 5-µg- cefotaxime disk [32]. These tests have excellent performances, but their main pitfalls are limited sensitivity, the requirement of an overnight incubation and doesn’t give information about the resistance mechanisms or genotype, and thus does not provide any information about the possible threats of spreading.

Detection of ESBLs at the genetic level represents an interesting alternative but remains costly, requires expertise and costly equipment, and may give false positive results due to the detection of inactive or incomplete resistance genes in a specimen [30]. Other techniques, such as matrix-assisted laser desorption ionization–time of flight mass spectrometry (MALDI-TOF MS) [33], are being developed, but they do require expensive equipment, a significant degree of expertise, and the kits are expensive. Finally, biochemical tests have been developed, namely, the Rapid ESBL NDP [34] test, and two commercially available tests: the β-Lacta test (BioRad), and the Rapid ESBL Screen [35]. The ESBL NDP test and the Rapid ESBL Screen are aimed at detecting ESBL producers, while the β-Lacta test is aimed at detecting not only ESBL producers but also cephalosporinase- and carbapenemase-producers. The sensitivity and specificity for detecting ESBL producers (*n* = 60) were 95% and 100% for the Rapid ESBL NDP test (after 2 h), 92% and 83% (after 2 h) for the Rapid ESBL Screen, and 88% and 71% for the β-Lacta test (after 15 min), respectively [36].

The NG-Test CTX-M MULTI detected all the CTX-M-expressing strains without ambiguous interpretation and showed 100% sensibility and specificity on standard agar containing plates used in clinical microbiology laboratories. Isolates representing the most prevalent variants of the five groups of CTX-Ms were detected, suggesting that this assay may be used on all the continents, irrespective of the prevalence of the different CTX-M variants present.

It is assumed that early identification of ESBL-E bacteraemia may lead to optimized treatment, contributing to better outcomes and early implementation of infection control measures [37]. The ß-Lacta test has been validated for rapid detection of Ambler class A ESBL-E from smudge plates prepared from positive blood cultures [38,39,40]. Even though the detection of expanded-spectrum cephalosporin (ESC)-resistant Enterobacterales was excellent, it required 2–3 h of growth prior to testing. Alternatively, Dortet et al. has shown that the ESBL NDP test may be able to detect ESBL-E within 30 min after a few centrifugation steps of positive blood cultures [34]. The NG-Test CTX-M MULTI was able to detect 10 out of 11 ESBL-producers in our study in less than 15 min.

Since the first description of *Kluyvera* as the progenitor of CTX-M-encoded β-lactamases with no or little phenotypic expression, as compared to their plasmid-borne counterparts [41], chromosomal counterparts belonging to the five sub-groups of acquired CTX-M β-lactamases have been found in *Kluyvera* spp. [7,29]. Even if *Kluyvera* spp. is considered as a benign saprophyte of gastrointestinal, urinary and respiratory tracts, it has been sporadically reported as the cause of clinically significant diseases at multiple anatomic sites, showing an ability to act as an opportunistic pathogen [42,43]. The NG-Test CTX-M MULTI was positive in only 32% (9/28) of the *Kluyvera* sp. isolates tested. In one case, the signal was strong, and in 8 cases the signal was weak, which correlates well with the observed resistance phenotype. The detection of the chromosomally encoded CTX-M-variants present in *Kluyvera* spp., is difficult as the expression level of these enzymes in these species is variable and very low [44].

*K. oxytoca* clinical isolates were also used as controls since they produce structurally related but chromosomally encoded ESBLs (OXY/K1) [45]. Hyperproduction of these chromosomally encoded class A OXY-β-lactamases confers resistance to aztreonam and expanded spectrum cephalosporins (ESCs) with the exception of ceftazidime and cephamycins, but OXY-2-variants conferring high-level resistance to ceftazidime have also been described [45]. The NG-Test CTX-M MULTI does not cross-react with *K. oxytoca* isolates, unless a plasmid-encoded CTX-M is also produced.

ESBLs, especially CTX-M-type are the main source of cephalosporin resistance in Enterobacterales of human origin in France. The NG-Test CTX-M MULTI is thus well adapted to the French epidemiology, and thus, its routine application of rapid diagnostic tests for ESBL-E detection should lead to improved antimicrobial management, particularly by reducing inappropriate use of carbapenems, especially for bacteraemia. Although these results are promising, they should be further confirmed in other countries where the prevalence and the epidemiology of ESBL-producers might be different and where pAmpC are more prevalent. In addition, more variants need to be tested, even though the variants tested correspond to most prevalent variants worldwide, they represent only 13% (29/229) of the known variants [28]. Further developments including, TEM-, SHV-, minor-ESBLs and the main pAmpCs (CMY, DHA) would improve the test to reach almost 100%.

## 5. Conclusions

The NG-Test CTX-M MULTI assay detected all the CTX-M-expressing strains without ambiguous interpretation and showed 100% sensitivity and specificity on standard agar containing plates used in clinical microbiology laboratories. Isolates from the 5 groups of CTX-Ms were detected either from bacterial cultures or directly from positive blood cultures. The NG-Test CTX-M MULTI assay is a commercially-available robust assay, which is stable for >24 months without refrigeration, user-friendly (no need for trained staff nor equipment for the readout), rapid (less than 15 min) and cost-effective ~7€ per test, as compared to commercially available molecular tests (ranging from 20 to >100 euros for BioFire-type PCRs). In settings where CTX-Ms are the major source of ESCs resistance, these tests should lead to improved antimicrobial management, particularly by reducing inappropriate use of carbapenems.

## Figures and Tables

**Table 1:** Evaluation of NG-Test CTX-M MULTI results on different culture media.

		Test Results on Culture Medium ^a^				
Bacterial Species	β-Lactamase	MH	COH	Uri-4	DRIG	ChromID
*K. pneumoniae*	CTX-M-15	P ^b^	P	P	P	P
*E. coli*	CTX-M-1	P	P	P	P	P
*E. coli*	CTX-M-14	P	P	P	P	P
*E. coli*	CTX-M-9	P	P	P	P	P
*E. coli*	CTX-M-8	P	P	P	P	P
*K. pneumoniae*	CTX-M-8	P	P	P	P	P
*E. coli*	CTX-M-2	P	P	P	P	P
*K. pneumoniae*	CTX-M-2	P	P	P	P	P
*E. coli*	SHV-12	N	N	N	N	N
*E. coli*	TEM-24	N	N	N	N	N
*E. coli*	KPC-2	N	N	N	N	N

^a^ MH, Mueller Hinton agar; COH, Columbia agar plus 5% horse blood; Uri-4, URISelect 4 agar medium; DRIG, Drigalski agar; ChromID, ChromID^TM^ ESBL agar; ^b^ P, positive result; N, positive result.

**Table 2:** Prospective evaluation of NG-Test CTX-M MULTI.

Species	Sample Origin	ESBL Identified	No. of Isolates	NG-Test CTX-M MULTI Results ^a^
*E. coli*	Urine	CTX-M-1	4	P
*E. coli*	Bile	CTX-M-1	1	P
*E. coli*	Urine	CTX-M-14	6	P
*E. coli*	Bile	CTX-M-14	1	P
*E. coli*	blood	CTX-M-14	1	P
*E. coli*	Urine	CTX-M-15	18	P
*E. coli*	blood	CTX-M-15	3	P
*E. coli*	Urine	CTX-M-27	2	P
*E. coli*	Urine	CTX-M-32	1	P
*E. coli*	Urine	CTX-M-55	1	P
*E. coli*	blood	CTX-M-9	1	P
*K. pneumoniae*	blood	CTX-M-14	1	P
*K. pneumoniae*	blood	CTX-M-15	25	P
*K. pneumoniae*	Urine	CTX-M-127	1	P
*K. pneumoniae*	blood	CTX-M-15	13	P
*K. pneumoniae*	blood	Hyper SHV-28	2	N
*E. cloacae*	Urine	CTX-M-15	7	P
*E. cloacae*	Urine	CTX-M-9	2	P
*E. cloacae*	blood	CTX-M-15	6	P
*E. cloacae*	blood	CTX-M-3	1	P
*E. cloacae*	blood	SHV-12	1	N
*C. koseri*	Urine	CTX-M-1	1	P
*C. koseri*	Urine	CTX-M-14	1	P
*Salmonella* sp.	blood	CTX-M-9	1	P
*C. freundii*	blood	CTX-M-15	1	P

^a^ P, positive result; N, negative result.

## Supplementary Materials

The following are available online at https://www.mdpi.com/2075-4418/10/10/764/s1, Table S1: Retrospective evaluation of NG-Test MULTI on well characterized isolates, Table S2: Retrospective evaluation of NG-Test MULTI on *K. oxytoca* isolates, Table S3: Retrospective evaluation of NG-Test MULTI on *Kluyvera* spp. isolates.
