# Supplementary material for: A Lateral Flow Immunoassay for the Rapid Identification of CTX-M-Producing Enterobacterales from Culture Plates and Positive Blood Cultures
Source: Diagnostics (Basel). 2020 Sep 28;10(10):764. doi: 10.3390/diagnostics10100764 (PMC7600033; doi:10.3390/diagnostics10100764)
Supplement: Supplementary file 1 [file diagnostics-10-00764-s001.pdf]

diagnostics

# A lateral flow immunoassay for the rapid identification of CTX-M-producing Enterobacterales

Sandrine BERNABEU[1,2], Kayaththiry C. RATNAM,[2] Camille GONZALEZ,[2] Hervé BOUTAL[3], Anaïs VOGEL[3], Karine DEVILLIERS[3], Marc PLAISANCE[3], Saoussen OUESLATI[1,2], Surbhi MALHOTRA-KUMAR[4,5], Laurent DORTET[1,2,6], Stéphanie SIMON[3], Hervé VOLLAND[3], Thierry NAAS[1,2,6*]

[1] Team Resist, UMR1184, Université Paris-Saclay - INSERM - CEA, LabEx Lermit,

[2] Bacteriology-Hygiene unit, APHP, Hôpital Bicêtre, Le Kremlin-Bicêtre, France

[3] Service de Pharmacologie et Immunoanalyse (SPI), CEA, INRA, Laboratoire d'Etudes et de Recherches en Immunonalyse, Université Paris-Saclay, F-91191, Gif-sur-Yvette, France

[4] Laboratory of Medical Microbiology, Vaccine & Infectious Disease. Institute, University of Antwerp, Antwerp, Belgium

[5] Members of ESCMID Study Group for Antimicrobial Resistance Surveillance - ESGARS

[6] Associated French National Reference Center for Antibiotic Resistance: Carbapenemase-producing Enterobacteriaceae, Le Kremlin-Bicêtre, France

*Supplemental tables 3*

Table S1: Retrospective evaluation of NG-Test MULTI on well characterized isolates

| β-lactamase content | CTX-M group[a] | Species | No. of isolates | NG-Test CTX-M Multi |
|---|---|---|---|---|
| CTX-M-1 | 1 | *C. freundii* | 1 | **P** |
| CTX-M-1 | 1 | *C. koseri* | 1 | **P** |
| CTX-M-1 | 1 | *E. coli* | 2 | **P** |
| CTX-M-1 + TEM-1 | 1 | *E. coli* | 1 | **P** |
| CTX-M-10 | 1 | *E. coli* | 1 | **P** |
| CTX-M-101 | 1 | *E. coli* | 1 | **P** |
| CTX-M-15 | 1 | *E. coli* | 1 | **P** |
| CTX-M-15 | 1 | *K. oxytoca* | 1 | **P** |
| CTX-M-15 | 1 | *K. pneumoniae* | 1 | **P*** |
| CTX-M-15 + SHV-27 | 1 | *K. pneumoniae* | 1 | **P** |
| CTX-M-15 + TEM-1 | 1 | *C. freundii* | 1 | **P** |
| CTX-M-15 + TEM-1 | 1 | *E. coli* | 1 | **P** |
| CTX-M-15 + TEM-1 | 1 | *K. oxytoca* | 1 | **P** |
| CTX-M-15 + TEM-1 | 1 | *M. morganii* | 1 | **P** |
| CTX-M-15 + TEM-1 | 1 | *P. mirabilis* | 1 | **P** |
| CTX-M-15 + TEM-1 + SHV-11 | 1 | *E. cloacae* | 2 | **P** |
| CTX-M-15 + TEM-1 + SHV-12 | 1 | *E. cloacae* | 1 | **P** |
| CTX-M-15 + TEM-1 + SHV-28 | 1 | *C. freundii* | 1 | **P**[#] |
| CTX-M-182 | 1 | *E. coli* | 1 | **P** |
| CTX-M-3 + TEM-1 | 1 | *E. coli* | 1 | **P** |
| CTX-M-3 + TEM-1 + SHV-11 | 1 | *K. pneumoniae* | 1 | **P** |
| CTX-M-32 + TEM-1 | 1 | *E. coli* | 1 | **P** |
| CTX-M-37 | 1 | *E. coli* | 1 | **P** |
| CTX-M-55 | 1 | *E. coli* | 2 | **P** |
| CTX-M-57 | 1 | *E. coli* | 1 | **P** |
| CTX-M-71 | 1 | *E. coli* | 1 | **P** |
| CTX-M-71 | 1 | *P. mirabilis* | 1 | **P** |
| CTX-M-82 | 1 | *E. coli* | 1 | **P** |
| CTX-M-127 | 1 | *K. pneumoniae* | 1 | **P** |
| CTX-M-2 + TEM-1 | 2 | *E. coli* | 1 | **P** |
| CTX-M-2 + TEM-1 + OKP6 | 2 | *K. pneumoniae* | 1 | **P** |
| CTX-M-100 | 25 | *E. coli* | 1 | **P** |
| CTX-M-94 | 25 | *E. coli* | 1 | **P** |
| CTX-M-8 | 8 | *E. coli* | 1 | **P** |
| CTX-M-8 + TEM-1 + SHV-28 | 8 | *K. pneumoniae* | 1 | **P**[#] |
| CTX-M-13 | 9 | *E. coli* | 1 | **P** |
| CTX-M-14 + TEM-1 | 9 | *E. coli* | 3 | **P** |
| CTX-M-17 | 9 | *E. coli* | 1 | **P** |
| CTX-M-18 | 9 | *K. pneumoniae* | 1 | **P**[#] |
| CTX-M-19 | 9 | *K. pneumoniae* | 1 | **P** |

| | | | | |
|---|---|---|---|---|
| CTX-M-24 | 9 | *E. coli* | 1 | **P** |
| CTX-M-27 | 9 | *E. coli* | 2 | **P** |
| CTX-M-65 | 9 | *E. coli* | 1 | **P** |
| CTX-M-9 | 9 | *E. cloacae* | 1 | **P** |
| CTX-M-93 | 9 | *E. coli* | 1 | **P** |
| SHV-12 | x | *E. coli* | 1 | N |
| SHV-12 + TEM-1 | x | *K. oxytoca* | 1 | N |
| SHV-12 + TEM-1 | x | *C. freundii* | 1 | N |
| SHV-28 + TEM-3 | x | *K. pneumoniae* | 1 | N |
| SHV-2a | x | *E. coli* | 1 | N |
| SHV-2a | x | *K. pneumoniae* | 1 | N |
| TEM-24 | x | *K. aerogenes* | 2 | N |
| TEM-3 | x | *E. coli* | 1 | N |
| TEM-3 | x | *E. cloacae* | 1 | N |
| TEM-52 | x | *E. coli* | 1 | N |
| TEM-52 + TEM-1 | x | *P. mirabilis* | 1 | N |
| VEB-1 | x | *E. cloacae* | 1 | N |

P, positive result; N, negative result

# Invalid result at the first attempt and detected as positive with a modified protocol

[a]1, CTX-M-1 group; 2, CTX-M-2 group; 8, CTX-M-8 group; 9, CTX-M-9 group; 25, CTX-M-25 group

Table S2: Retrospective evaluation of NG-Test MULTI on *K. oxytoca* isolates

| isolate # | Reference | Main acquired β-lactamase content | NG-Test CTX-M Multi |
|---|---|---|---|
| 1 | R8J10 | NDM-1 | N |
| 2 | R13E1 | GES-7 | N |
| 3 | O53G7 | CMY-2 | N |
| 4 | O60J4 | OXA-48 | N |
| 5 | O61I8 | OXA-48 + VIM-1 | N |
| 6 | 230C9 | OXA-48 | N |
| 7 | 230C10 | OXA-48 | N |
| 8 | 232E1- | OXA-48+ CTX-M-15 | **P** |
| 9 | 232F7 | OXA-48 | N |
| 10 | 233F2 | VIM-1 | N |
| 11 | 234C3 | VIM-1 | N |
| 12 | 235E9- | DHA-1 | N |
| 13 | 237F6 | OXA-48 + VIM-1+ CTX-M-15 | **P** |
| 14 | 243J9 | NDM-1 | N |
| 15 | B5R10 | NDM-1 | N |
| 16 | 34F& | NDM-1+ CTX-M-15 | **P** |
| 17 | O50H3 | VIM-1+ CTX-M-15 | **P** |
| 18 | 10C8 | CMY-2 | N |
| 19 | 2011-5 | TEM-1 | N |
| 20 | 2011-48 | TEM-1 | N |
| 21 | 2011-88 | TEM-1 | N |

P, positive result; N, negative result

Table S3: Retrospective evaluation of NG-Test MULTI on *Kluyvera* spp isolates

| Isolate # | Species | Identifier | Main acquired ß-lactamase content | NG-Test CTX Multi |
|---|---|---|---|---|
| 1 | *K. cryocrescens* | CIP7952 | None | P* |
| 2 | *K. ascorbata* | KJ8 | PER-2 | N |
| 3 | *K. ascorbata* | KJ68 | PER-2 | p* |
| 4 | *K. ascorbata* | CIP8295 | None | p* |
| 5 | *K. ascorbata* | CIP7953 | None | p* |
| 6 | *K. cryocrescens* | CIP8296 | None | p* |
| 7 | *K. cryocrescens* | CIP7954 | None | p* |
| 8 | *K. ascorbata* | Bud | None | p* |
| 9 | *K. ascorbata* | FL-1 | None | N |
| 10 | *K. ascorbata* | FL-2 | None | p* |
| 12 | *K. cochlae* | S3/1 49T | None | N |
| 13 | *K. cochlae* | S3/1 913 | None | N |
| 14 | *K. cochlae* | S3/1 859 | None | N |
| 15 | *Kluyvera* | spp1 | None | N |
| 11 | *K. georgiana* | O12H4 | None | N |
| 16 | *K. ascorbata* | O23C8 | None | **P** |
| 17 | *K. ascorbata* | O38I1 | None | p* |
| 18 | *K. ascorbata* | 18-276 | None | N |
| 19 | *K. ascorbata* | 20-142 | None | N |
| 20 | *K. ascorbata* | 20-141 | None | N |
| 21 | *K. ascorbata* | 20-147 | None | N |
| 22 | *K. ascorbata* | 20-10 | None | N |
| 23 | *K. ascorbata* | 20-9 | None | N |
| 24 | *K. ascorbata* | 20-46 | None | N |
| 25 | *K. ascorbata* | 20-58 | None | N |
| 26 | *K. ascorbata* | 20-60 | None | N |
| 27 | *K. ascorbata* | 20-69 | None | N |
| 28 | *K. ascorbata* | 20-71 | None | N |

**P**, positive result; p*, weak positive result; N, negative result
